# A Wonderful Journey: The Diverse Roles of Adenosine Deaminase Action on RNA 1 (ADAR1) in Central Nervous System Diseases

**DOI:** 10.1111/cns.70208

**Published:** 2025-01-03

**Authors:** Lin Cheng, Ziying Liu, Chunxiao Shen, Yinyi Xiong, Sang Yol Shin, Yong Hwang, Seung‐Bum Yang, Zhiying Chen, Xiaorong Zhang

**Affiliations:** ^1^ Department of Neurology Affiliated Hospital of Jiujiang University Jiujiang Jiangxi China; ^2^ Jiujiang Clinical Precision Medicine Research Center Jiujiang Jiangxi China; ^3^ Department of Pathology Affiliated Hospital of Jiujiang University Jiujiang Jiangxi China; ^4^ Department of Rehabilitation Affiliated Hospital of Jiujiang University Jiujiang Jiangxi China; ^5^ Department of Emergency Medical Technology Wonkwang University College of Medicine Iksan Jeonbuk‐do Republic of Korea; ^6^ Department of Emergency Medicine Wonkwang University College of Medicine Iksan Jeonbuk‐do Republic of Korea; ^7^ Department of Paramedicine Wonkwang Health Science University Iksan Jeonbuk‐do Republic of Korea

**Keywords:** ADAR1, Aicardi–Goutières syndrome, CNS diseases, innate immunity, neurodegenerative diseases, tumors

## Abstract

**Background:**

Adenosine deaminase action on RNA 1 (ADAR1) can convert the adenosine in double‐stranded RNA (dsRNA) molecules into inosine in a process known as A‐to‐I RNA editing. ADAR1 regulates gene expression output by interacting with RNA and other proteins; plays important roles in development, including growth; and is linked to innate immunity, tumors, and central nervous system (CNS) diseases.

**Results:**

In recent years, the role of ADAR1 in tumors has been widely discussed, but its role in CNS diseases has not been reviewed. It is worth noting that recent studies have shown ADAR1 has great potential in the treatment of neurodegenerative diseases, but the mechanisms are still unclear. Therefore, it is necessary to elaborate on the role of ADAR1 in CNS diseases.

**Conclusions:**

Here, we focus on the effects and mechanisms of ADAR1 on CNS diseases such as Aicardi–AicardiGoutières syndrome, Alzheimer's disease, Parkinson's disease, glioblastoma, epilepsy, amyotrophic lateral sclerosis, and autism. We also evaluate the impact of ADAR1‐based treatment strategies on these diseases, with a particular focus on the development and treatment strategies of new technologies such as microRNAs, nanotechnology, gene editing, and stem cell therapy. We hope to provide new directions and insights for the future development of ADAR1 gene editing technology in brain science and the treatment of CNS diseases.

## Graphical abstract

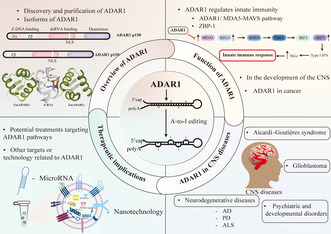

## 1 Introduction

With the continuous development of new technologies, the mysterious world of RNA modification is gradually being revealed. Researchers have already found approximately 170 different RNA modifications in living cells, with the vast majority of RNA modifications occurring in transfer RNAs (tRNAs) [1, 2]. However, recent excitement in this research field has been generated by the observation of RNA modifications in messenger RNAs (mRNAs). Adenosine (A) deamination to inosine (I) (A‐to‐I RNA editing) is one of the most abundant modifications in mRNA, with more than 100 million sites in the human genome [3].

ADAR1, an adenosine deaminase that acts on RNA, plays a pivotal role in RNA editing and is one of the most common enzymes for the A‐to‐I RNA editing of double‐stranded RNA (dsRNA) substrates [4, 5, 6] that plays an important role in maintaining the normal function of the central nervous system (CNS). For example, by regulating RNA editing, ADAR1 is involved in the maintenance of neuronal homeostasis, regulation of synaptic plasticity, and inhibition of innate immune activation [7, 8, 9, 10]. Its dysfunction or gene mutations can cause a range of CNS diseases, such as amyotrophic lateral sclerosis (ALS) [11], Aicardi–Goutières syndrome (AGS) [12, 13, 14], schizophrenia [15], suicidal depression [16], Alzheimer's disease (AD) [17], and Parkinson's disease (PD) [18]. Epidemiology data show that there are approximately 40 million people worldwide who are thought to have AD [19], and the number of people with PD is expected to increase by 9 million by 2030 [20], suggesting that ADAR1 dysfunction may contribute to a broad disease burden. While direct links between ADAR1 activity and these conditions require more empirical evidence, the intricate mechanisms through which ADAR1 exerts its effects in various CNS disorders remain a compelling domain for future investigations.

While the bulk of ADAR1‐related research has focused on its autoimmune implications and role in cancer [21], recent evidence also suggests that ADAR1 has far‐reaching regulatory implications for CNS homeostasis. In this account, we describe in detail the initial purification of ADAR1 and its structure, localization, and posttranslational modification. We focus on the function of ADAR1 in the CNS, and its role and molecular mechanism of action in diseases such as AD, ALS, AGS, schizophrenia, epilepsy, and PD. Finally, we discuss the significance of ADAR1 in the treatment of these CNS diseases and the latest developments in related treatment strategies, analyze the current research foci in the area, and discuss the questions that remain unaddressed.

## 2 Overview of ADAR Family

Adenosine deaminases acting on RNA (ADARs) are A‐to‐I RNA‐editing enzymes that catalyze the chemical conversion of adenosine to inosine in dsRNA substrates. In mammals, there are three ADAR proteins: ADAR1, ADAR2, and ADAR3 [22]. ADAR2 and ADAR3 have been extensively studied in the context of RNA editing and immune regulation within the CNS. However, this article primarily focuses on ADAR1, as it has been widely investigated in relation to tumors and immune‐related diseases, yet its role in the CNS remains underexplored.

### 2.1 Isoforms of ADAR1 and Their Functional Implications

ADAR1 was first identified in the late 1980s during studies on antisense RNA molecules in *Xenopus* oocytes that revealed its ability to convert adenosine (A) to inosine (I) within double‐stranded RNA (dsRNA) [23, 24]. This process, known as A‐to‐I RNA editing, is a major nucleotide modification that typically occurs in the structured or double‐stranded regions of RNA. In the human transcriptome, more than 10^6^ A‐to‐I RNA‐editing sites have been identified [3, 22]. This type of editing significantly affects many cellular processes, including protein coding and RNA folding, splicing, and turnover [25, 26, 27].

Studies have shown that ADAR1 has three distinct isoforms, the full‐length ADAR1 (p150) and two functionally active short isoforms (p110 and p80). These isoforms have different protein structures but are arranged into similar domains [28]. ADAR1p80, localized primarily in the nucleolus, lacks key domains due to the selective splicing of exon 2 [28, 29, 30]. There are very few studies on ADAR1p80, and its specific function is not clear, but it may be related to inflammatory stimulation [28]. This review focuses on the two major isoforms, ADAR1p150 and ADAR1p110, which are transcribed from different promoters and generated via alternative splicing [31, 32, 33].

**FIGURE 1:**
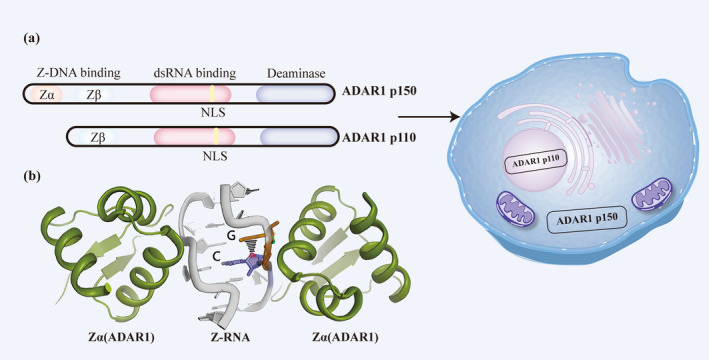
Structure of ADAR1. (a) Structure of ADAR1 and location of ADAR1. ADAR1 p150 is located in the cytoplasm, while ADAR1 p110 is mainly located in the nucleus. (b) The Zα domain of ADAR1p150 binds to Z‐RNA.

ADAR1p150 is an interferon‐inducible isoform predominantly localized in the cytoplasm [31, 32, 33]. ADAR1p150 contains two Z‐DNA‐binding domains (Zα and Zβ), three dsRNA‐binding domain repeats (dsRBD1‐3), and a unique deaminase domain [12, 34, 35]. The Zα domain, consisting of approximately 65 amino acids, is highly conserved and interacts specifically with the zigzag sugar‐phosphate backbone of Z‐DNA/Z‐RNA, contributing to the enzyme's high substrate specificity and binding affinity [36, 37, 38]. Zα enhances the enzyme's substrate specificity and activity in vitro and acts synergistically with the downstream Zβ domain [39]. The Zα domain of ADARp150 is critical for its proper binding of Z‐RNA substrates and is a key factor in the type I interferon (IFN) response pathway. Two common point mutations in the Zα domain (N173S and P193A) are a major cause of neurodegenerative diseases [36]. In contrast, ADAR1p110 has a truncated N‐terminus and lacks one Z‐DNA domain (Zα). ADAR1p150 and ADAR1p110 also have NLSs in the common dsRBD region (Figure 1) [40]. Although these two isoforms of ADAR1 have enzyme activity, their differential localization in cells and divergent structural characteristics suggests that their target RNA and biological functions are different [41, 42, 43]. Studies have shown that ADAR1p150 mainly plays a role in regulating immunity, while ADAR1p110 plays a role in the cellular stress response [44].

### 2.2 The Roles of ADAR2 and ADAR3 in the CNS

ADAR2 is most highly expressed in the brain, although it is also found in other tissues, and is critical for neurological function. Studies have shown that mice lacking ADAR2 die within 3 weeks of birth and develop severe seizure symptoms related to the effect of an increased influx of calcium ions through glutamate receptors on synaptic plasticity across the brain [25, 45]. ADAR3 expression is restricted to the CNS and is catalytically inactive [46], and in vitro studies have shown that ADAR3 may be involved in the regulation of ADAR1 and ADAR2 expression [47]. Together, these enzymes contribute to the complex RNA‐editing landscape in the CNS, and future studies are needed to explore their potential synergistic interactions and the implications for neurodegenerative and psychiatric disorders. Future studies should focus on exploring their specific synergistic effects or whether they have antagonistic effects.

## 3 Function of ADAR1

ADAR1 isoforms are essential for many biological functions, including innate immunity [48, 49, 50], cellular stress responses [51], hematopoiesis [52, 53, 54], nervous system function [33, 55, 56, 57], and cancer [58, 59]. The role of ADAR1 in innate immunity is well established, and it has been shown to be an important therapeutic target in cancer treatment [2]. However, few studies have found direct links between the function or dysfunction of ADAR1 and the specific mechanisms of neurodegeneration, which points to this being an area ripe for further exploration and discovery. Next, we briefly describe the role of ADAR1 in innate immunity and tumor therapy, focusing on the role of ADAR1 in the CNS and its possible mechanism of action, and provide ideas for the further exploration of ADAR1's role in the CNS.

### 3.1 ADAR1 Regulates Innate Immunity

ADAR1‐mediated A‐to‐I RNA editing is a critical mechanism for distinguishing self from nonself RNA [49, 60, 61]. This process marks endogenous dsRNAs as self‐molecules, preventing their recognition by RNA sensors, such as melanoma differentiation‐associated protein 5 (MDA5) and retinoic acid‐inducible gene I (RIG‐I), which can trigger innate immune responses [62]. ADAR1 has emerged as a critical regulator in the context of innate immunity, bridging the gap between the detection of endogenous and exogenous RNA molecules and the prevention of autoimmune responses [49, 63, 64, 65]. By editing dsRNA, ADAR1 disrupts duplex structures and suppresses inappropriate immune activation [66, 67]. Dysregulation of this pathway can result in autoinflammatory diseases and exacerbate CNS inflammation, a hallmark of many neurodegenerative diseases [4, 17, 51].

#### 3.1.1 ADAR1/MDA5‐MAVS Pathway

**FIGURE 2:**
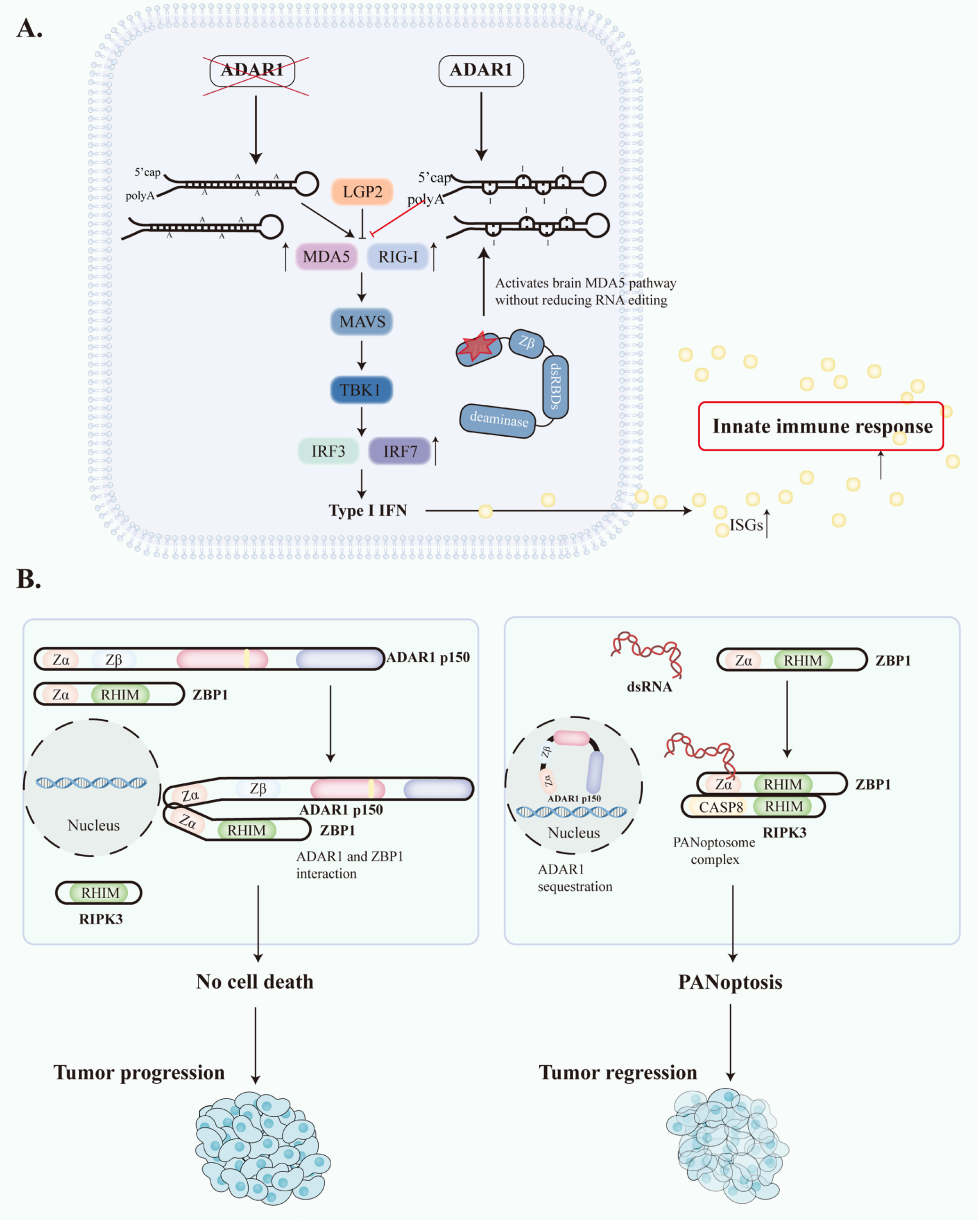
ADAR1/MDA5‐MAVS pathway and ADAR1 in tumor progression and regression. (A) Left: Without ADAR1, unedited dsRNA activates MDA5 and RIG‐I, leading to increased type I IFN and ISG expression. Right: ADAR1 edits dsRNA, preventing immune activation. Mutations in ADAR1's Zα domain activate the brain MDA5 pathway without reducing RNA editing. (B) ADAR1 inhibition mediates ZBP1‐based apoptosis in antitumor therapy. ADAR1 p150 and ZBP1 interact through the Zα domain to prevent the pan‐apoptosis of macrophages and promote tumor progression. When sequestration of ADAR1 p150 occurs in the nucleus, dsRNA accumulates in the cytoplasm. dsRNA recognition is via the Zα domain of ZBP1, which is then linked to RIPK3 via the RHIM domain. Eventually, PANoptosome complexes are formed, which trigger PANoptosis and thus inhibit tumor progression.

ADAR1 is involved in mediating innate immunity via the classic ADAR1/MDA5‐MAVS/TBK1/ISGs pathway [49, 68, 69, 70]. When ADAR1‐editing activity is lacking, the overexpression of the RNA sensors RIG‐I and MDA5 is induced, activating the downstream effectors MAVS and TBK1 and the transcription factors IRF3 and IRF7. These factors increase the expression of type I IFN and IFN‐stimulated genes (ISGs), leading to the induction of innate immune responses [49] (Figure 2). This aberrant immune activation may be associated with CNS disease, as IFNs can reach the CNS from the periphery and regulate the function of not only microglia and astrocytes but also neurons and oligodendrocytes to have major effects on cognition and behavior [71]. Importantly, mutations in the Zα domain of ADAR1p150 can independently activate MDA5‐dependent pathways in the CNS without reducing the overall amount of RNA editing, underscoring the complexity of ADAR1's immune regulatory functions [69].

#### 3.1.2 ZBP‐1 Is Involved in the Innate Immune Regulation Function of ADAR1

Recent studies have shown that ZBP‐1 is also an important molecule in ADAR1's regulation of innate immunity. Numerous studies have confirmed that there is a regulatory connection between ADAR1 and ZBP‐1 [12, 63, 72, 73, 74]. Mutations in the Zα domain of ADAR1 result in defective RNA editing, leading to the accumulation of Z‐RNA and activation of ZBP1 [18]. The combined knockout of caspase‐8 and RIPK3 or caspase‐8 and MLKL was reported to aggravate the pathogenic effect of the ADAR1p150 mutation. Many studies have confirmed that the activation of ZBP1 elicits caspase‐8‐dependent apoptosis and MLKL‐mediated necroptosis of ADAR1‐deficient cells [68, 75, 76, 77]. In addition, loss of ADAR1 function activates ZBP1‐mediated autoimmune disease and embryonic death, suggesting that ADAR1 is negatively associated with immune activation [66, 68, 75]. The interplay between ADAR1 and ZBP1 has emerged as a fundamental aspect of innate immunity's regulatory landscape. However, there are still many open questions that require further investigation.

### 3.2 ADAR1 in Cancer

In recent years, a large number of studies have increasingly linked ADAR1 to cancer progression, and major pro‐oncogenic effects are being seen in a growing number of cancer types, including hepatocellular carcinoma [78], lung cancer [79, 80], breast cancer [81, 82, 83], gastric cancer [84], cervical cancer [85], colorectal cancer [86, 87], thyroid cancer [88, 89], and glioblastoma (GBM) [90, 91].

The role of ADAR1 in cancer is very complex and context‐dependent, involving not only its A‐to‐I editing function but also its immune regulatory functions [40]. ADAR1's involvement in the editing of cancer‐promoting mechanisms mainly includes coding edits [88], targeting the 3′UTR [92], binding to or editing the hairpin intermediates of miRNAs [93], and mediating splicing regulation [88]. For example, ADAR1 can directly interact with Dicer independently of its catalytic activity and RNA‐binding activity, promote the processing of cancer‐promoting miRNAs, and play a role in promoting cancer [93]. ADAR1 acts as a pro‐oncogenic protein independently of its active deaminase domain. In GBM, METTL3 targets ADAR1 through a novel molecular pathway, METTL3/ADAR1/CDK2, resulting in the overexpression of the ADAR1 protein, which promotes GBM progression independently of its editing mechanisms [90].

ADAR1 is not only associated with the treatment of tumors through immunomodulatory effects but also with the development of tumors. ADAR1 inhibits inflammatory cell death by interacting with the Zα2 domain of ZBP1, thereby limiting the interaction between ZBP1 and RIPK3 and promoting tumorigenesis (Figure 2). Although current research on the relationship between ADAR1 and cancer is making significant progress, the specific mechanism still needs to be further explored to provide new targets and ideas for future tumor treatment strategies.

### 3.3 ADAR1 Is Involved in the Development of the CNS

ADAR1 not only has a central role in innate immunity and tumor development but is also necessary for growth and development [94, 95]. Mice that lack the *Adar1* gene perish at the embryonica stage [55, 96, 97]. In this part, we focus on ADAR1's role in CNS development, in which it has been identified as a particularly critical player, functioning through editing mechanisms that posttranscriptionally modify RNA transcripts and influencing neuronal differentiation, maturation, and the overall neural developmental landscape. Chen et al. [55] demonstrated that an ADAR1 deficiency in human embryonic stem cells considerably affected their differentiation and neural induction. The study clearly showed that ADAR1 is crucial for the normal progression of stem cells toward a differentiated neural fate, providing direct evidence of ADAR1's role in CNS development. Further unraveling the complexity of ADAR1's role, research by Behm et al. [98] revealed an interesting dynamic in which the accumulation of nuclear ADAR2, regulated in part by ADAR1, influences A‐to‐I RNA editing during neuronal development. ADAR1 was found to be involved in the editing of serotonin 5‐hydroxytryptamine subtype 2C receptor pre‐mRNA in neural tissue and to be related to neuron survival [99, 100]. In addition, recent studies have shown that ADAR1 plays an important role after nerve injury, especially under pathological conditions such as ischemic stroke. One study found that ADAR1 can promote the proliferation of activated astrocytes after ischemic stroke and induce a series of inflammatory responses by producing reactive oxygen species (ROS). These responses further lead to neuronal apoptosis and promote the progression of nerve injury [6]. This suggests that ADAR1 has critical roles not only during neural development but also in damage repair and inflammatory responses in the CNS. By regulating the activity of ADAR1, it may provide new targets and strategies for the treatment of neurological diseases such as ischemic stroke. Collectively, these studies have provided a compelling narrative on ADAR1's integral role in CNS development. By regulating RNA‐editing processes, ADAR1 influences the gene expression patterns essential for neural differentiation and maturation. The promising research directions suggested by these studies present fertile ground for further exploration, with the potential to unlock novel therapeutic strategies for neurodevelopmental disorders. The close association between ADAR1 and many CNS diseases is discussed in more detail in the following sections.

## 4 ADAR1 in CNS Diseases

The function of ADAR1 in the CNS is garnering increasing attention, with its role in various neurological diseases being particularly notable. ADAR1 regulates immune responses through its RNA‐editing function, preventing endogenous RNA from triggering inappropriate immune activity, which is crucial for maintaining normal CNS function [4]. In this section, we explore the role of ADAR1 in several major CNS diseases, including AGS, neurodegenerative diseases, and psychiatric and developmental disorders. By understanding the mechanisms of ADAR1's activity in these diseases, we can better comprehend the pathological processes as well as identify new directions and targets for future therapeutic strategies.

### 4.1 Aicardi–Goutières Syndrome

AGS is a severe autoinflammatory disorder [101] in which ADAR1 plays a key role. In the pathophysiology of AGS, serious loss of ADAR1 function can occur [39], and the brain experiences severe inflammation because of innate immune activation. Almost all individuals affected by AGS show severe nerve dysfunction [102]. Common clinical AGS manifestations include a progressive loss of cognitive ability, the degradation of intelligence, cramps, dystonia, and movement disorders [103, 104, 105].

*ADAR1* is one of several genes associated with AGS. ADAR1 regulates immune responses in cells through its catalytic activity, largely by editing dsRNA to prevent it from triggering the activation of the IFN pathway [8, 9]. RNA editing is required for innate immune tolerance, as unedited cellular RNA is recognized as “nonself” by RLRs, particularly MDA5, which triggers IFN pathway activation [43, 60]. The elevated expression of type I IFN and ISG is commonly observed in the CNS and peripheral blood cells of AGS patients [106, 107], and IFN signaling pathway activation is thought to play a central role in brain pathogenesis [108, 109]. According to current interpretations, ADAR1 variation in AGS causes a failure in immunity due to reduced dsRNA editing [56, 110]. This notion has been supported by several mouse models, such as K999N and D1113H mutant mice, in which even partial loss of RNA‐editing activity leads to the MDA5‐dependent activation of dsRNA‐induced signaling [111, 112]. *Adar1*‐knockout mouse models showed markedly enhanced type I IFN signaling and elevated peripheral autoimmune responses [113]. In addition, the expression of type I IFN and ISG was significantly upregulated in the embryonic cells of *Adar1*‐knockout mice [52, 60]. These results support the interpretation that unedited endogenous RNA triggers the activity of the RNA sensor MDA5, leading to the initiation of the IFN response. In addition, these findings reinforce the theory that the RNA‐editing activity of ADAR1 is critical for innate immune homeostasis.

**FIGURE 3:**
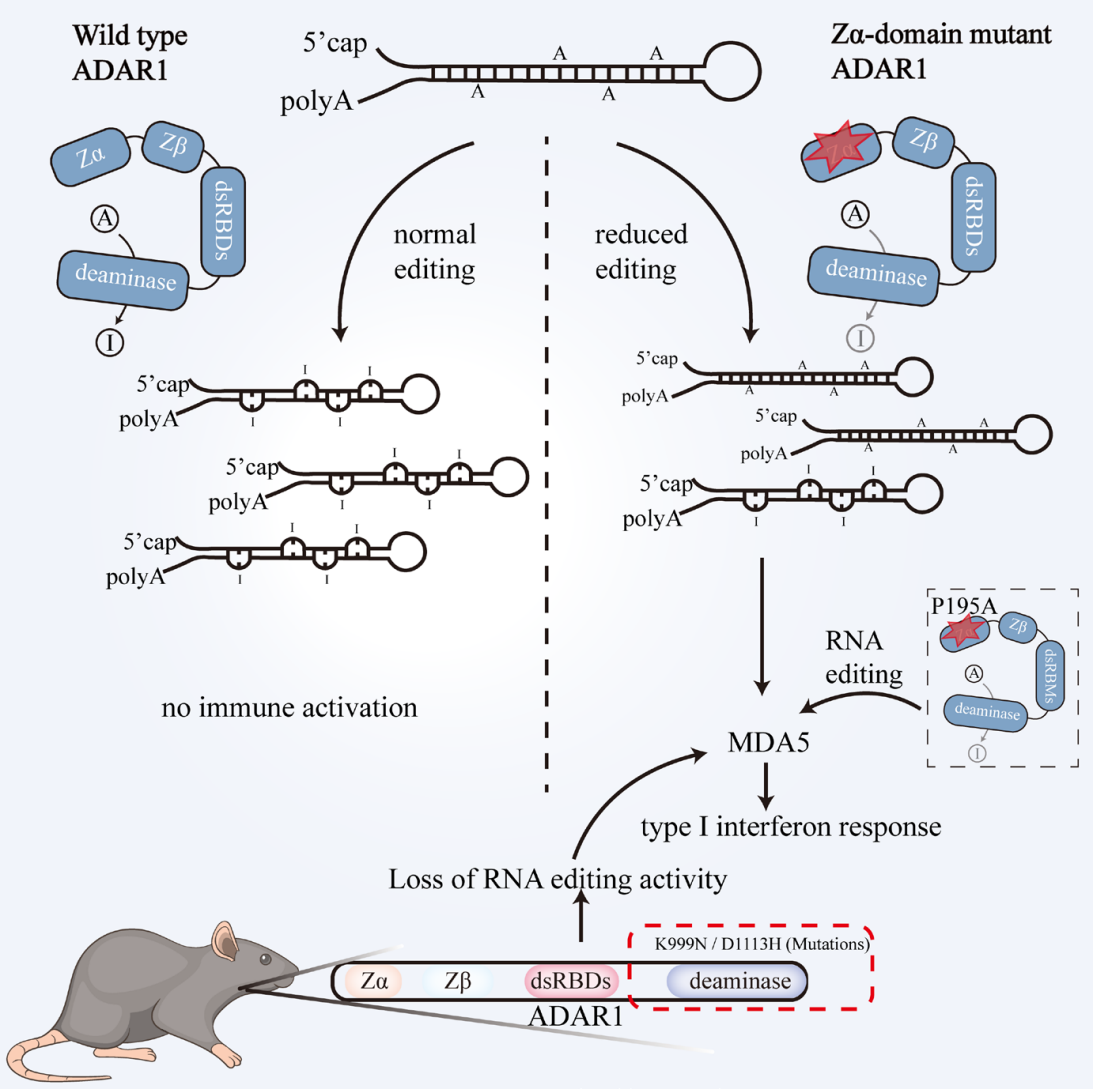
Role of ADAR1 in AGS pathogenesis and immune activation. Mutations in ADAR1, particularly in the Zα domain, disrupt RNA editing, leading to the recognition of cellular RNA as “nonself” by MDA5. This recognition triggers a type I IFN response, causing the severe neuroinflammation and autoinflammatory responses characteristic of AGS. Mouse model studies further support the critical role of ADAR1's RNA‐editing activity in maintaining innate immune tolerance and preventing excessive immune activation.

ADAR1 combines with various dsRNA forms, including typical type α and β Z‐RNAs. ADAR1 Zα domain mutations are common in patients with AGS [39]. Guo et al. [114] analyzed P195A mutant mice carrying mutations affecting the ADAR1 Zα structural domain and found the specific mutations did not affect the overall RNA‐editing activity of ADAR1 but activated MDA5 of the RNA‐sensing signal pathway, leading to strong ISG expression in the brain. Moreover, de Reuver and Maelfait [12] found that mutations in the ADAR1 Zα domain led to the accumulation of dsRNA and triggered an MDA5‐mediated IFN‐I response. The interaction between Z‐RNA and ADAR1 can prevent the development of MDA5/MAVS‐dependent type I IFN responses (Figure 3) [39]. Furthermore, ADAR1 blocks the activation of pathogenic type I IFNs by the endogenous Z‐RNA‐dependent activation of ZBP1, suggesting that ZBP1 may be a key molecule in the type I IFN diseases caused by ADAR1 mutations [68, 75, 115]. ADAR1 can prevent autoinflammation by inhibiting spontaneous ZBP1 activation [68]. All these results provide a theoretical basis for the development of therapeutic approaches in the future. Studying ADAR1 has not only revealed its key roles in the pathogenesis of AGS but also provided important clues to understanding the immunological basis of other CNS diseases. These findings help to deepen our understanding of the relationship between neurological dysfunction and provide new directions for the development of therapeutic strategies in the future.

### 4.2 Neurodegenerative Diseases

Neurodegenerative diseases are a group of heterogeneous neurological disorders that adversely affect the lives of millions of people worldwide and involve the progressive loss of neurons in the CNS or peripheral nervous system [116]. Classic neurodegenerative diseases include AD, PD, and ALS [117]. The pathophysiological mechanism of degenerative diseases is extremely complex and includes apoptosis, oxidative stress, protein aggregation, neuroinflammation, and other possible pathogenic factors [118, 119, 120, 121]. However, there is also a strong association between necrosis and neurodegeneration and neuroinflammation [118]. The generation of ROS can cause neuronal damage and simultaneously release a series of molecules that activate microglia and astrocytes. In turn, these activated glial cells release proinflammatory cytokines, triggering inflammation and further exacerbating neuronal damage [119]. The accumulation of β‐amyloid (Aβ) oligomeric peptide and tau protein in AD, and the accumulation of α‐synuclein (α‐syn) in PD, can lead to neuroinflammation, neurotransmitter imbalance, dendritic morphology changes, and synaptic damage, and these mechanisms together promote the occurrence and progression of neurodegeneration [120, 121, 122]. The pathological process of neurodegenerative diseases relies on interactions among multiple factors, and inflammation is particularly closely related to other pathological mechanisms. Therefore, it can be said that neuroinflammation is a major mechanism of brain aging and age‐related neurodegenerative diseases such as ALS [123] and is characterized by the proinflammatory activation of glial cells [124]. Astrocytes are the most common glial cells in the brain and are tightly integrated with neurons and other CNS cells [125]. Experiments show that ADAR1 and glial fibrillary acidic protein expression levels are negatively correlated [17], which suggests that ADAR1 changes significantly influence the functions of astrocytes throughout the brain. These findings were further validated through in vitro experiments on primary human astrocytes. The experiments involved suppressing ADAR expression using siRNA knockdown or treatment with an ADAR inhibitor (8‐azaadenosine), both of which resulted in increased levels of the proinflammatory cytokine interleukin 6 and intercellular adhesion molecule 1 (a marker of proinflammatory astrocytes) [126]. ADAR1 acts as a potential neuroinflammatory regulator by controlling the accumulation of dsRNA, which is a proinflammatory activator. Studies have shown that ADAR1 may stimulate type I IFN inflammatory signaling by regulating transposable‐element‐derived dsRNA and that reductions in ADAR1 could lead to neuroinflammation in AD [4, 17]. This preliminary study has laid the foundation for future studies on the role of ADAR1 in ALS and other neurodegenerative diseases. For example, the activation of neuroinflammation in AD is closely related to the overactivation of astrocytes and microglia [127, 128]. As a dsRNA‐editing enzyme, ADAR1 shows decreased expression in the brain of AD patients, and this is accompanied by increased levels of transposon‐derived dsRNA [17], which are thought to stimulate the type I interferon signaling pathway through receptors such as RIG‐I or MDA5, further aggravating neuroinflammation [129]. More importantly, transcriptome analysis has shown that the increase in TE transcripts in AD patient brains occurs mainly in Alu and LINE‐1 (L1) sequences, which are major editing targets of ADAR1 [17, 67, 130, 131]. This TE‐derived double‐stranded RNA accumulation directly activates inflammatory pathways and may also lead to further synaptic plasticity damage and the destruction of the blood–brain barrier by affecting the extracellular matrix function of astrocytes, thus aggravating the neuropathological process of AD [17, 132, 133, 134]. An imbalance in the ADAR1 regulation process may be an important driver of the inflammatory cascade in AD. Similarly, as discussed above, inflammation plays a significant role in the pathophysiology of PD and ALS [135, 136, 137]. It is reasonable to hypothesize that ADAR1 may act as a potential neuroinflammatory regulator in these neurodegenerative diseases by controlling the accumulation of dsRNA [17, 67, 130, 131]. Through this mechanism, ADAR1 could influence the progression of PD, ALS, and other neurodegenerative disorders. Although current research in this area remains limited, this theory has plausibility and warrants further investigation. Exploring the role of ADAR1 in these diseases may provide valuable insights and open new avenues for therapeutic development.

ADAR1 plays multiple roles in neurodegenerative diseases, exerting an impact on their development through the neuroinflammatory pathway and other mechanisms. ADAR1 may contribute to the astrocyte‐mediated neuronal apoptotic process by increasing the inflammatory responses produced by ROS stress [6]. In addition, ADAR1 plays a key role in inhibiting endogenous RNA sensing and regulates the degree of innate immune system activation, which in turn induces corresponding tissue damage [138]. Association studies into neurodegenerative diseases have shown that the abnormal expression of ADAR1 is closely related to the pathogenesis of such diseases, highlighting its importance in the maintenance of nervous system health. These findings provide new perspectives and a deeper understanding of the pathological mechanisms of neurological diseases while providing new targets and directions for the future development of therapeutic strategies against these diseases.

### 4.3 Psychiatric Disorders

Bipolar disorder (BD) is a common psychiatric disorder characterized by fluctuations in mood between depression and mild mania or full‐blown manic episodes [139]. The role of inflammation in this disorder has been confirmed [140]. As already stated, ADAR1‐mediated RNA editing is a key mechanism in the regulation of inflammatory responses [60]. Experimental evidence indicates that a set of RNA‐editing‐based blood biomarkers, including PDE8A, CAMK1D, GAB2, IFNAR1, KCNJ15, LYN, MDM2, and PRKCB, can be used in diagnosing BD with high sensitivity and specificity [141]. Studies into ADAR1‐mediated RNA‐editing functions suggest that measuring ADAR1 variations at the peripheral level may allow for the stratification of patients with BD. The results have been promising, and the role of ADAR1 in psychiatric disorders is gradually being revealed, thus further experiments in this area are warranted [142].

### 4.4 Developmental Disorders

Autism spectrum disorder (ASD) is a highly heterogeneous neurodevelopmental disorder characterized by deficits in communication and social behavior [143]. The etiology of ASD includes congenital immune dysfunction, evidence of neuroinflammation, and the activation of microglial cells [144]. ADAR1‐mediated A‐to‐I editing is widespread in the brain, with editing levels gradually increasing during development [145, 146], but the extent of miRNA editing in ASD remains largely unknown. To comprehensively identify miRNA editing sites in ASD, researchers analyzed the miRNA‐seq profiles of brain samples from ASD patients and normal controls. The analysis revealed significantly elevated editing levels of hsa‐mir‐376a‐1_9_A_g in ASD patient brain samples. Thus, A‐to‐I editing of miR‐376a‐5p may lead to neurodevelopmental abnormalities by suppressing GPR85 and NAPB [147]. ADAR1's role in regulating the expression of genes related to RNA editing suggests it may be an influential factor in the development of ASD.

### 4.5 Glioblastoma

A GBM is a type of very malignant, incurable CNS tumor with considerable cell heterogeneity and a high degree of aggressiveness [148]. GBMs account for about 49% of malignant brain tumors; they show considerable resistance to standard radio‐ and chemotherapies and surgical excision and have a poor prognosis [149, 150]. GBMs, being one of the most aggressive and lethal forms of brain cancer, necessitate the discovery and analysis of novel targets for effective treatment strategies. Previous research has suggested that ADARs are closely related to GBMs. For example, ADAR2 is expressed at low levels in GBMs, and the low levels of ADAR2 editing lead to malignant phenotypes [151]. In contrast, ADAR1 is highly expressed in many tumors, including GBMs. A study by Tassinari et al. [90] unveiled an editing‐independent mechanism by which ADAR1 promotes oncogenesis. The research highlighted METTL3, known to be upregulated in GBMs, as a methylator of ADAR1 mRNA. The study showed METTL3 enhances ADAR1 protein levels through an editing‐independent mechanism and, in turn, contributes to a pro‐tumorigenic mechanism. In another study that found a link between ADAR1 and GBM pathology, Jiang et al. [91] revealed how ADAR1‐mediated RNA editing intersects with ganglioside catabolism to support GBM stem cell (GSC) maintenance. The study elucidated the role of ADAR1 in promoting GSC self‐renewal and stemness, thereby spotlighting a potential weakness in GBM defenses that could be leveraged for therapeutic intervention. Research has showcased ADAR1 as a critical component in the complexity and resilience of GBMs and offered novel insights and possible avenues for therapeutic interventions. Through editing‐dependent and independent mechanisms, ADAR1 plays pivotal roles in GBM pathology.

## 5 Mechanisms of ADAR1 Activity in CNS Pathology

### 5.1 Immune Regulation

ADAR1 plays a crucial role in preventing intracellular antiviral dsRNA sensor activation, which has major implications for CNS disease. ADAR1 knockout in mice causes extensive liver damage, leading to embryonic death [97, 152]. In mouse embryos at the molecular level, ADAR1 defects result in the activation of innate immune responses characterized by the excessive production of IFNs and the induction of ISG expression [52]. These observations strongly implicate ADAR1 in the control of innate immunity. dsRNAs are produced during the replication cycle of DNA and RNA viruses [153] but are also abundantly encoded in mammalian genomes; therefore, surveillance mechanisms are needed to distinguish “self” from “nonself” [154]. A‐to‐I RNA editing of dsRNAs acts as a self‐marker to prevent the aberrant activation of antiviral cytoplasmic dsRNA sensor proteins [65]. Mammalian cells express a variety of innate immune receptors, including MDA5, PKR, and ZBP1, that function in combination with dsRNA and dsRNA activation [12]. Of all tested nucleic acid sensor IFN pathway components in the cytoplasm, only knocking out dsRNA sensor MDA5 or its downstream effectors MAVS was reported to prevent the lack of a functional ADAR1 causing mouse embryonic death [60, 155, 156, 157]. Loss of ADAR1 expression in adult mice results in lethal MDA5‐dependent and MAVS‐dependent autoinflammation [43, 156]. A study confirmed in a mouse model completely lacking A‐to‐I editing of ADAR1 and ADAR2 [158]. Hu and others reported that ADAR1 uses different mechanisms to prevent endogenous dsRNA activation. MDA5 and PKR, both innate immune sensors, competitively edit dsRNAs, and ADAR1 can prevent MDA5 activation as well as inhibit PKR activation [49]. The complexity of this mechanism shows that ADAR1's role in maintaining the immune steady‐state and prevent autoimmune reactions is very important, and its dysfunction can cause serious illness associated with the CNS [159]. In addition, we previously described that ADAR1 can induce neuronal apoptosis by inducing the secretion of IL‐1β, IL‐6, and TNF‐α by astrocytes through the production of ROS [6]. Given that the adult brain consists of multiple regions, each containing unique neuronal subtypes and morphologically distinct combinations of astrocytes, it is possible that astrocytes exhibit regional specialization [160]. For example, hippocampal astrocytes show greater gap junction coupling and potassium ion currents compared to striatal astrocytes, while striatal astrocytes possess larger territories but fewer neuronal interactions [161]. These observations suggest that ADAR1 may differentially regulate CNS functions in distinct brain regions by interacting with regionally specialized astrocytes and their unique responses to oxidative stress and inflammatory signaling. In the future, further studies can be conducted to determine whether ADAR1 functions differently in astrocytes from different regions to fully understand its regional and cell type‐specific roles in the CNS. Furthermore, given the functional diversity of neuron subtypes, such as glutamatergic and GABAergic neurons, future studies should explore whether ADAR1's activity in RNA editing and immune modulation exhibits specificity in regulating these neuron–astrocyte interactions across different brain regions. These studies have revealed the crucial role of ADAR1 in regulating the innate immune system and have provided potential therapeutic targets for the future treatment of CNS diseases. Through in‐depth studies of ADAR1 and its mechanisms of interaction with the immune system, we can develop new treatment methods to prevent or alleviate nerve inflammation in neurodegenerative diseases and related CNS diseases caused by the dysfunction of ADAR1.

### 5.2 RNA‐Editing Features

Increasing evidence suggests that posttranscriptional RNA modifications play important roles in the complex functions of the CNS [162]. Many ARAD1 A‐to‐I‐edited RNAs are being found in the mammalian and human CNS and can alter the function of translated proteins, including neurotransmitter receptors and ion channels. Thus, a role for dysregulated RNA editing in the pathogenesis of neurological diseases has been postulated [163]. Various studies have shown that these RNA‐editing sites have important functions in neural development and other biological processes [164]. As our understanding of the function of noncoding sequences and their impact on human diseases deepens, the role of the RNA‐editing function of ADAR1 in disease development is attracting increasing attention [165]. At present, evidence shows that CNS‐related diseases, such as AGS [166], bilateral striatal necrosis [167] and acute spinal cord injury [168, 169], are related to the RNA‐editing function of ADAR1. A‐to‐I RNA editing leads to changes in certain functions of ion channel determinants and neurotransmitter receptors in vertebrates and invertebrates [10]. This suggests ADAR1 RNA editing can affect neurotransmitter receptors and is vital for synaptic transmission and plasticity, the ability of synapses to strengthen or weaken over time, which is critical for learning and memory [7]. RNA‐editing disorders of neurotransmitter receptors are associated with a variety of diseases of the nervous system, highlighting the importance of ADAR1's editing activity in maintaining the normal function of the CNS. These findings suggest that the RNA‐editing function of ADAR1 is not only critical in maintaining normal neural function in the CNS but is also a key player in the pathogenesis of a variety of neurological diseases. Further studies of ADAR1's RNA‐editing activity and its regulatory mechanisms will provide important clues to understanding the pathological processes of complex diseases and may also help with the development of new treatment strategies.

## 6 Therapeutic Implications

### 6.1 Potential Treatments Targeting ADAR1 Pathways

ADAR1 is involved in the RNA‐editing process and is associated with neurodevelopment in the CNS, making it a promising target for regulating AGS and other neurodegenerative diseases. Many ADAR1 knockout [96, 170, 171] and mutant mouse models, including P195 A [51], E861 A [156], K948 N [172], and Y177 A [110], are conducive to the exploration of ADAR1's mechanisms of action and finding targeted therapy strategies for related diseases. The ADAR1 mutations associated with AGS mainly include the W197 mutation [56] and the P195 mutation in mice [21], but the latest study in this area identified a new AGS‐related mutation by CRISPR/Cas‐9 technology, Adar D1113H [112]. This mutation causes an IFN‐stimulated gene to be preferentially and potently expressed in the brain and causes astrocyte and microglial hyperplasia neuroinflammatory responses. Ellagic acid (EA), a phenolic small‐molecule inhibitor of RNase L, can inhibit RNase L ribonuclease activity and consequently attenuate ADAR1‐associated apoptosis, indicating that EA is a viable therapeutic target for AGS with ADAR1 mutations [173]. ADAR1's role in RNA editing and immune regulation makes it a promising target for neurodegenerative and autoimmune diseases. However, the therapeutic strategies for targeting ADAR1 in the CNS and peripheral systems may differ due to the unique challenges presented by the blood–brain barrier (BBB) and the regional specialization of ADAR1 activity. For example, while ADAR1‐mediated dsRNA editing can suppress neuroinflammation in CNS diseases such as AGS [8, 9], its dysregulation in peripheral tissues may contribute to autoimmune diseases and cancers [174, 175]. Mouse models, such as Adar D1113H and Adar1 W197A/W197A, have revealed that blocking RNA‐sensing pathways through MDA5 or LGP2 deletion can mitigate ADAR1 mutation‐induced neuroinflammation and brain lesions [55, 112, 176]. These findings provide insights into potential CNS‐specific therapeutic strategies, including targeted modulation of the MDA5/MAVS pathway to reduce neuroinflammation without triggering peripheral immune dysfunction [55].

However, caution is warranted, as ADAR1 inhibitors developed for CNS diseases may influence peripheral immune responses, potentially affecting tumor immunosurveillance. ADAR1 has been shown to play dual roles in cancer, where its RNA editing promotes tumor growth and immune evasion [174, 175]. A high‐throughput virtual screening study identified lithospermic acid and regal side B as potential small‐molecule inhibitors of ADAR1p150 that target the Zα domain and can inhibit protein translation and arrest tumor cell proliferation and thus exert antitumor effects [177]. Recent research shows that rebecsinib is a potential clinical ADAR1p150 antagonist [178]. However, these inhibitors must be carefully evaluated to ensure that they do not exacerbate CNS inflammation or impair neural functions.

### 6.2 Some Other Targets or Technology Related to ADAR1

#### 6.2.1 MicroRNA

MicroRNAs (miRNAs) are known to have very important roles in CNS diseases [179, 180]. Li, Lei, and Sun [181] introduced the concept that miRNAs are involved in a critical layer of posttranscriptional regulation relevant to ADAR1 function. The intersection between miRNA activity and ADAR1‐mediated RNA editing is a noteworthy area for potential therapeutic interventions, with the multifaceted regulatory mechanisms that could be leveraged against neurodegradation. Various studies have confirmed the close relationship between microRNA and ADAR1 [182, 183, 184]. For example, a study in HeLa cells showed that miR‐3614‐5p represses the p110 and p150 forms of ADAR1 and reduces constitutive and IFN‐induced A‐to‐I RNA editing [185]. Future research should focus on elucidating whether therapeutic strategies targeting the interaction between miRNAs and ADAR1 could enable CNS‐specific interventions while mitigating off‐target effects in peripheral tissues.

#### 6.2.2 Nanotechnology

In an exploration of the frontier of therapeutic innovations, Wei et al. [186] investigated methods at the convergence of nanotherapeutic and stem cell strategies in CNS diseases. Their study highlighted the therapeutic potential of leveraging these advanced treatment modalities along with ADAR1's modulation activity to enhance their efficacy. Ding et al. [187] developed a genetically engineered nanovesicle (siAdar1‐LNP@mPD1) that silences ADAR1 expression and overexpresses PD1. This engineered nanovesicle led to increased IFN‐β/γ production and made cancer cells more sensitive to IFN‐β/γ, inducing considerable cell growth arrest, while blocking the PD‐L1 immune checkpoint to exert strong antitumor immunity. The nanovesicles can deliver therapeutic drugs, such as small molecules, to specific types of cells or tissues, increasing the local concentration of therapeutics and minimizing side effects to realize targeted drug activity [188, 189]. The application of this technology can also solve the contradiction in ADAR1 expression differences in CNS diseases and tumors. These systems can simultaneously target CNS inflammation and peripheral tumorigenesis by tailoring drug delivery to tissue‐specific requirements. Advances in nanotechnology could address the challenges posed by ADAR1's diverse roles in different systems, allowing for precise therapeutic modulation. Although various obstacles and challenges remain, nanotechnology therapy brings hope for those with CNS diseases and tumors related to ADAR1; however, continuous improvement and development, from laboratory experiments to the clinical application stage, are necessary.

In summary, while direct research on therapeutic strategies targeting ADAR1 in CNS diseases remains nascent, the existing literature on the various mechanisms and therapeutic approaches, including nanotherapeutics, stem cell therapies, and microRNAs provides a rich resource for understanding how modulating ADAR1 activity could contribute to novel treatments. Future research should aim to directly investigate ADAR1's role and the impact of its therapeutic modulation within the complex pathophysiology of CNS diseases to harness its full potential as a target for intervention.

## 7 Conclusion and Prospects

The multiple functions of ADAR1 in cells complicate our understanding of its role in the CNS. Through RNA binding, RNA editing, and protein interactions that mediate its activity, ADAR 1 exerts extensive control over the global transcriptome. In the CNS, it is involved not only in the development of the nervous system but also in a variety of CNS diseases, making it a potential target for treatment. Although our understanding of the biology of ADAR1 has expanded rapidly in recent years, particularly in the treatment of tumors, our comprehension of its role in CNS diseases remains limited. This includes neurodegenerative diseases such as AD, PD, and ALS, as well as psychiatric disorders. Further research is needed to enhance our understanding of ADAR1's role in these conditions. It is worth noting that the combined application of ADAR1 with nanotechnology, miRNA, and stem cell technology could bring major changes to the treatment of neurological diseases. However, how to balance the absence of ADAR1 in the CNS with its high expression level in tumors is a matter of concern, especially in patients with CNS disease and concomitant tumors. A more complete ADAR1‐targeting strategy would likely produce the broadest and most robust response but would increase the risk of side effects in normal tissues. The key to the development of anti‐ADAR1 therapeutics will be to find the right balance for an optimal therapeutic.

## Author Contributions

The initial idea for this manuscript was from Seung‐Bum Yang, Zhiying Chen and Xiaorong Zhang; Lin Cheng, Ziying Liu and Chunxiao Shen wrote and revised the manuscript; Yinyi Xiong, Sang Yol Shin and Yong Hwang edited the manuscript; Seung‐Bum Yang, Zhiying Chen and Xiaorong Zhang read, reviewed, and approved the final manuscript. All authors have read and agreed to the published version of the manuscript.

## Conflicts of Interest

The authors declare no conflicts of interest.

## Data Availability

Data sharing not applicable to this article as no datasets were generated or analyzed during the current study.
